# Supplementary material for: The Human–Animal Relationship as the Focus of Animal-Assisted Interventions: A One Health Approach
Source: Int J Environ Res Public Health. 2019 Sep 29;16(19):3660. doi: 10.3390/ijerph16193660 (PMC6801464; doi:10.3390/ijerph16193660)
Supplement: Supplementary file 1 [file ijerph-16-03660-s001.pdf]

INTERSPECIFIC GLOSSARY

Interspecific relationship: the interaction between individuals of different species. It is defined through the parameters of the content of the interaction, the quality, frequency, identity, and roles of the contractors, the motivations, the purpose, and the means [1].

Harmonic interspecific relationship: a relationship in which the human referent represents a secure base for the animal according to the principles of secure attachment and where the animal does not respond to the replacement needs of the human. It is a relationship in which reciprocity is realized. This type of relational dimension gives, in particular, the dog, an important sense of security, which is necessary for the animal to best express itself and neutralize the stress of the first meeting until the next need for adaptation. The veterinary handler, within the therapies and healthcare facilities, or animal handler, allows the animal to move away and even step back during the session [2-5].

Reciprocity: reciprocity is what happens between two people, two things or two groups when an action or object received from one of the two parties corresponds to an action or an equivalent object from the other party. The interspecific reciprocity is, therefore, a very complex system of relational feedback that initiates with bodily gestures and attitudes and the activation of emotional sense–motor models between the two species. It is a relational condition in which respect, attention to the other party, authenticity, and congruence are central [6].

Interspecific communication: a communicative exchange between individuals of different species. Human communication is a natural process of transmitting ideas, information, emotions, and feelings from one person to another within a certain amount of time [7]. It consists of 7% verbal language, 38% para-verbal (tone, volume, speed of speech), and 55% non-verbal (facial expressions, positions, movement, clothing, etc.). Interspecific communication is realized through analogical communication (para-verbal and nonverbal) [7-8].

Complex system: a system composed of many components which may interact with each other. Such a system can be represented by a network where nodes represent the components and links represent their interactions [9]. The term "complex" derives from the Latin verb *complector*, meaning to wrap and keep tightly wound, and in a metaphorical sense, to embrace, comprehend, unite everything in itself. The etymology of the word is useful for eliciting the thought that is at the base of the theory [10,11], according to which the approach to a complex system cannot be understood if one proceeds with an analytical view through a simple breakdown, trying to examine every single part. A complex system can only be explained if you try to obtain a vision of the whole, verifying how much the single parts influence each other reciprocally [12].

Reference

1. Rogers, C.R. *Psicoterapia di Consultazione* Ed. Astrolabio - Ubaldini, Roma, Italia; 1971; ISBN: 883400115X
2. Prato-Previde, E.; Custance, D.M.; Spiezio C. and Sabatini, F. Is the dog–human relationship an attachment bond? An observational study using ainsworth's strange situation. *Behaviour* **2003**, *140*, 225-254, doi:10.1163/156853903321671514.
3. Topál, J.; Miklósi, A.; Csányi, V.; Dóka, A. Attachment behavior in dogs (Canis familiaris): a new application of Ainsworth's (1969) Strange Situation Test. *J. Comp. Psychol.* **1998**, *112*, 219-229.
4. Solomon, J.; Beetz, A.; Schöberl, I.; Gee, N.; Kotrschal, K. Attachment security in companion dogs: adaptation of Ainsworth's strange situation and classification procedures to dogs and their human caregivers. *Attach. Hum. Develop.* **2019**, *21*, 389–417. https://doi.org/10.1080/14616734.2018.1517812
5. Hausberger, M.; Roche, H.; Henry, S.; Visser, E.K. A review of the human–horse relationship. *Applied Anim. Behav. Sci.* **2008**, *109*, 1-24. doi:10.1016/j.applanim.2007.04.015

6. Fenelli, A.; Volpi, C.; Guarracino, E.; Galli, V.; Esposito, M. The reciprocity rule in bord construction is as the reading key in relational. *Riv. Psich.* **2011**, *46*, 298. doi: 10.1708/1009.10975.
7. Runcan P.L.; Constantineanu, C.; Ielics, B.; Popa, D. The Role of Communication in the Parent-Child Interaction. *Proc. Soc. Behav. Sci.* **2012**, *46*, 904 – 908.
8. Jeannin, S.; Gilbert, C.; Leboucher, G. Effect of interaction type on the characteristics of pet-directed speech in female dog owners. *Anim. Cogn.* **2017**, *20*, 499-509. doi: 10.1007/s10071-017-1077-7.
9. Fortunato, S. Reuven Cohen and Shlomo Havlin: Complex Networks. *J. Stat. Physics* **2011**, *142*, 640–641. doi:10.1007/s10955-011-0129-7. ISSN 0022-4715.
10. Morin E. (1993), Introduzione al pensiero complesso, Milano, Sperling & Kupfer.
11. Morin E. (1985). "Le vie della complessità". in G.Bocchi, M.Ceruti (a cura di) "La sfida della complessità". Milano, Feltrinelli, pag. 49-60.
12. Menna, L.F. The scientific approach to Pet therapy. The Method and Training according to the Federiciano Model, 1st ed.; University of Naples Federico II: Napoli, Italia, 2018; 979-12-200-3991-8.
